# A scoping review of the use of music as an arts-based method in migrant health research

**DOI:** 10.12688/hrbopenres.13817.1

**Published:** 2023-12-06

**Authors:** Fran Garry, Anne MacFarlane, Sylvia Murphy Tighe, Pattie Punch, Helen Phelan

**Affiliations:** 1 Irish World Academy of Music and Dance and Health Research Institute (HRI), University of Limerick, Limerick, County Limerick, V94 T9PX, Ireland; 2 Irish World Academy of Music and Dance, Participatory Health Research Unit, University of Limerick, Limerick, V94 T9PX, Ireland; 3 Participatory Health Research Unit, School of Medicine and Health Research Institute (HRI), University of Limerick, Limerick, V94 T9PX, Ireland; 4 Department of Nursing & Midwifery and Health Research Institute (HRI), University of Limerick, Limerick, County Limerick, V94 T9PX, Ireland; 5 Department of Nursing & Midwifery, University of Limerick, Limerick, V94 T9PX, Ireland; 6 Glucksman Library, Faculty of Arts, Humanities & Social Sciences, University of Limerick, Limerick, County Limerick, V94 T9PX, Ireland; 7 Irish World Academy of Music and Dance and Health Research Institute (HRI), University of Limerick, Limerick, County Limerick, V94 T9PX, Ireland

**Keywords:** Music, arts-based research, migrant health, scoping review

## Abstract

**Background:**

There is a growing awareness of the need to include people’s lived experiences in health decision-making. While much progress has been made in this field, exclusionary patterns persist regarding migrant participation in health research. The aim of this scoping review was to examine the available literature around the use of music as an arts-based research (ABR) method in migrant health research in order to extend knowledge of creative methods and tools used for migrant participation in health research.

**Methods:**

Our review follows a scoping review methodology. Searches were conducted in 11 electronic databases between June and August, 2020. We identified 14 eligible references published between January 2009 and August 2020. We analysed how music is utilised as an arts-based research method in community-based health and wellbeing contexts primarily with refugees, asylum seekers, undocumented migrants, and members of resettled immigrant communities.

**Results:**

The findings show that music’s role is most prominent as a tool for recruitment and engagement followed by its use as a tool for dissemination. Music is effective as a means to co-produce and communicate knowledge of lived experiences of migration and related wellbeing issues.

**Recommendations and conclusions:**

Our recommendations for further research include the need for increased detail on the musical element in ABR projects; Explicit identification of such research as ABR; Greater recognition of a multi-arts understanding of music in the context of ABR; Harnessing the potential of music in ABR across the research cycle. We conclude that arts-based research using music shows promise for capturing the complexity of migrants’ lives and health issues in an ethical way. It warrants further investigation in empirical studies in multiple clinical and community settings to understand its processes and impacts on the evidence base for migrant health.

## Introduction

It is becoming increasingly important to incorporate public involvement in health decision-making. This is recognised by policy makers, academics and community stakeholders for multiple and inter-related reasons such as the need for greater accountability of health agencies to the public, the need for public engagement to build a comprehensive evidence base and contribute to its implementation ( Gibson *et al*., 2012; Popay *et al*., 2021). It is increasingly recognised that people are experts in their own health and that this experiential knowledge must be incorporated into health research contexts ( Popay & Williams, 2006; Wallersteins *et al*., 2018. This knowledge, based on lived experience, can play an important role in positive health promotion ( Ascenso *et al*., 2018; Tarr *et al*., 2014); the prevention of ill health ( Chabot *et al*., 2019; Fancourt & Perkins, 2018; the management and treatment of illness ( Cao *et al*., 2016; Wan *et al*., 2010); health services and strategy development ( WHO, 2018; WHO Europe, 2002; WHO Europe, 2018), and health research ( HRB Strategy, 2016–2020; Wallerstein *et al*., 2018).

Despite significant progress regarding public involvement in health research, further development is needed in specific areas, particularly in terms of the need to increase migrant participation in health research ( de Freitas & Martin, 2015; MacFarlane, 2019; MacFarlane *et al*., 2021). However, this is both complex and challenging for various reasons. For example migrants are a heterogeneous group of peoples. There is no agreed singular definition for a ‘migrant’. The term has been most broadly defined as “anyone who has moved from their habitual residence (within or across borders)” ( https://www.iom.int/who-is-a-migrant). Migration and mobility can also lead to major challenges in terms of social inclusion and research participation where the reason for migration, transit journey and arrival conditions in a host country are not favourable (e.g. for people seeking protection, or who are undocumented) ( Roura *et al*., 2021). Furthermore, belonging to an ethnic group with particular social and cultural practices may further undermine social inclusion and research participation within migrant groups or host populations ( Roura *et al*., 2021; Rubin *et al*., 2010). The incorporation of social and cultural practices in research contexts is the key focus of our review. Considering the ethical imperatives of incorporating migrants’ expertise in the development of a broader knowledge-base, a key question in gathering the available evidence of best practice in this context, is how migrant involvement in health research can be optimised in ways that are socially and culturally attuned with their lives and health needs?

A Participatory Health Research paradigm prioritises active engagement of participants in the research process, from inception to completion and dissemination, and has relevance to the exploration of this question. Participatory Health Research emphasises the co-creation of knowledge and community consultation, action and ownership ( Wright, 2015). There are multiple approaches and methodologies in the ‘family’ of Participatory Health Research: many of them draw attention to the ways in which spaces are set up for community members and other, usually more powerful, actors from the academy, health sector or government to meet and work together ( MacFarlane, 2019). Participatory spaces are shaped by the tools, practices and techniques that are used by researchers/facilitators to create and support mutual dialogue between these actors from diverse backgrounds ( Cornwall & Coelho, 2006; de Freitas & Martin, 2015; Kothari, 2001; MacFarlane, 2019; Power, 2001). In music research, for example, it has been shown that musical practices, particularly singing, are effective in creating dialogic space ( Marsh, 2019), fostering community participation and engagement by evoking empathy ( Ahlquist, 2006; Bithell, 2014; Clarke *et al*., 2015; Magowan, 2019; Phelan, 2017) accelerating social bonding in newly formed and diverse singing groups ( Pearce *et al*., 2015), and creating a sense of belonging for newly arrived migrant people ( Balsnes, 2016; Phelan, 2017; Phelan *et al*., 2017; Raanaas *et al*., 2019). Literature with a specific focus on music and migrant health and wellbeing in community settings is also increasing ( Balsnes, 2016; Cain *et al*., 2020; Henderson *et al*., 2017; Phelan, 2017; Phelan *et al*., 2017).

While music’s role in health and wellbeing is well documented, it is notable that reviews on arts-based methods in health contexts illustrate a lack of published research regarding the use of music as an arts-based research method ( Boydell *et al*., 2012; Coemans & Hannes, 2017; Fraser & al Sayah, 2011). Thus it is important to know how experiential knowledge from participatory music-making spaces underpinned by values of trust, inclusion, and raising participant voices ( Bartleet & Higgins, 2018; Higgins & Shehan Campbell, 2010; Welch *et al.*, 2014) can be harnessed in the research cycle of arts-based research projects. How can knowledge about music’s capacity to foster empathy and enhanced understanding of human experience in intercultural community projects ( Bartleet *et al*., 2009; Burnard *et al*., 2018; Howell, 2018; Li & Southcott, 2012; Southcott & Joseph, 2015) particularly in the context of migration ( Balsnes, 2016; Magowan, 2019; Marsh, 2019; Phelan, 2017; Raanaas *et al*., 2019) inform and improve research processes and outcomes?

Therefore, a scoping review which focuses specifically on the *use of music as an arts-based method in the field of migrant health* is necessary. The use of artistic practices as a component of research design is regularly labelled as arts-based research (ABR). Leavy (2009) defines ABR as “a set of methodological tools used by qualitative researchers across the disciplines during all phases of social research, including data collection, analysis, interpretation and representation […] arts-based practices draw on literary writing, music, performance, dance, visual art, film and other mediums” (p.ix). Research can be represented in multiple artistic forms encompassing visual arts, literary arts, and performing arts which include music, singing and dance ( Leavy, 2009 pp. 2–3).

This scoping review required attention to existing evidence across diverse disciplines that are not typically studied together: ABR, music, migrants, and health. This is needed to facilitate learning across disciplines and identify gaps in current knowledge. This can be used as a foundation to build ethically guided, effective strategies for using music as a participatory, arts-based method in migrant health research.

The aim of this scoping review was to examine the available literature around the use of music as an arts-based research (ABR) method in migrant health research in order to extend knowledge of creative methods and tools used in participatory projects involving migrants in health research. The objectives were to:

- Ascertain the extent of current publications using music in migrant health research and identifying itself as ABR.
- Identify the research stages (participating, generating data, analysing, interpreting, disseminating) in which music features as an ABR method or part thereof.
- Identify whether particular musical practices are used more than others as part of the research design and implementation.
- Identify the key strengths and challenges discussed in the literature around the use of music as a research tool in migrant health research.
- Identify gaps in current knowledge and use these as a foundation to build effective strategies towards increasing access to and knowledge of participatory, arts-based methods using music in migrant health research ( Garry *et al*., 2020, p.5).

## Methods

We formed an inter-disciplinary team with expertise in music research (authors 1 and 5), nursing (author 3), primary healthcare (author 2), migrant health (authors 1,2,3 and 5), and a librarian (author 4).

A scoping review was identified by us as the most appropriate methodology to address the broad nature of our research question and objectives. A scoping review can be used to identify and map key concepts and types of available evidence in order to provide an overview of a particular field of research ( Arksey & O’Malley, 2005; Levac *et al*., 2010; Munn *et al*., 2018). This review was guided by Arksey and O’Malley’s (2005) framework, and includes updated recommendations by Levac *et al*. (2010) and the JBI Reviewer’s Manual ( Peters *et al.*, 2020). Stages include: (i) Identifying the research question; (ii) Identifying relevant studies; iii) Study selection; (iv) Charting the data; (v) Collating, summarising, and reporting results; and (vi) Consultation. The objectives, inclusion criteria and methods for this scoping review were specified in advance and documented in a published protocol ( Garry *et al*., 2020) which can be accessed online at: https://doi.org/10.12688/hrbopenres.13121.1. This review is reported in line with the PRISMA-ScR guidelines ( Garry *et al.*, 2023).

### Identifying the research question

The research question guiding this scoping review is: “What is known about music as an arts-based method in migrant health research?” The identification of the research question involved an iterative process of searching and revising our key search terms with the aim of capturing the most pertinent literature relevant to our research goal. We collectively identified four core concepts encompassing the interdisciplinary focus of this scoping review. These are: ABR, music, migrants, and health. We specifically explored how music is utilised as an ABR method in the context of migrant health and wellbeing ( Garry *et al*., 2020).

### Identifying relevant studies

***Eligibility criteria.*** All studies such as journal articles, books, book chapters and grey literature that integrated music in the research design or implementation, and self-identified as arts-based research, or incorporated music-making with migrant people(s); and physical, mental or social wellbeing were eligible for inclusion. Publications had to be in the English, and published between 2009 and 2020. The publication of Leavy’s definition of arts-based research in 2009 was the basis of our review ( Leavy, 2009). All members of the research team contributed to discussions regarding eligibility criteria at the commencement of the scoping review ( Garry *et al*., 2020).

***Search strategy.*** The terminology used in our search strategy was developed in order to identify the most appropriate body of literature in line with our research question and objectives ( Arksey & O’Malley, 2005). The design of our search strategy involved interdisciplinary knowledge sharing and joint decision-making by the research team around the definitions, concepts, and terminology involved ( Levac *et al*., 2010). In order to test the suitability of search terms, preliminary searches were conducted in Taylor & Francis Online and CINAHL, searching article titles, abstracts, keywords and subject headings relating to our four key concepts: ABR, music, migrants and health. Our search strategy was further developed in collaboration with a senior faculty librarian. Our final search strategy included a comprehensive set of keywords (see Table 1: Key Search Terms; and the full search strategy as *Extended data*; Garry *et al.*, 2023) which were adapted across all databases encompassing the broad fields of arts, social science and health. Our strategy included checking the reference lists of included papers and relevant book chapters in order to identify any potential eligible publications not retrieved in the initial database searches.

**Table 1.** Key search terms.

Arts-Based Research	Music	Migrant	Health
“Arts-Based Research” OR “arts-informed research” OR “arts practice research”	Music OR “Music listening” OR “Music practice” OR Singing OR Songs OR “Choral music” OR Choirs OR “Solo music” OR “Instrumental music” OR “Ensemble music” OR Songwriting OR “Musical performance”	asylum* OR refugee OR migrant OR migrat* OR emigrant OR emigrat* OR immigrant OR nomad OR foreigner OR displaced OR stateless OR state-less OR noncitizen OR non-citizen OR outsider OR newcomer OR “newly arrived” OR “new arrival” OR “recent entrant” OR “non national” OR non-national	Health OR Wellbeing OR “Well being” OR Well-being OR “Physical health” OR “Mental health” OR “Physical wellbeing” OR “Mental wellbeing” OR “Social wellbeing” OR “Quality of life”

Electronic database searches were conducted in Scopus, Medline, Web of Science, CINAHL, ProQuest Academic Search Complete, Social Science Premium Collection, APA PsycINFO, Springerlink, Taylor & Francis Online, ProQuest Dissertations and Theses A&I. We used Google Advanced as a back-up search to identify any additional literature that was not already included through library database searching. Searches were conducted between June and August, 2020. The search terms were developed in relation to our core concepts to reflect the diversity of types of migrants and music forms, and a holistic understanding of health ( Garry *et al*., 2020).

### Study selection

Following completion of the search, two reviewers independently screened the studies by title and abstract (authors, 1 and 3) followed by full-text reviews guided by our inclusion and exclusion criteria. Any uncertainties or disagreements regarding inclusion or exclusion of sources were resolved by a third reviewer (author 5). Eligible studies were managed using Mendeley reference manager.

### Charting the data

Data were extracted using an MS Excel spreadsheet. Charting elements and complementary questions included JBI recommended classifications such as publication details, and general study details such as aims/purpose, methodological design, study population, methods and methodological design ( Peters *et al*., 2020). Additional categories, specific to our review, were used to guide the data extraction process. These included: definitions of ABR, forms of music, stages of the research where music was employed, details of migrant groups, research settings, study teams, ethical issues, health focus, evaluation of music, and limitations/quality issues. These additional charting elements supported the iterative process of collating, summarising, and reporting the results in line with our key question and objectives ( Garry *et al*., 2020). One reviewer completed the data charting process, which was fully verified by a second reviewer (authors 1 and 5).

### Collating, summarising and reporting the results

As recommended by the JBI Reviewer’s Manual ( Peters *et al*., 2020), a narrative summary mapping the findings from the extracted data complements the charted results and is presented here in line with our key question and objectives. We engaged in qualitative thematic construction ( Elo & Kyngas, 2008; Levac *et al*., 2010) through a process of identifying and grouping similar qualitatively described ( Sandelowski, 2000) charted information to provide an overview of the literature in line with the purpose of the review. Findings were organised into thematic categories such as aims/purpose, methodological design, key findings about ABR, music, migrants and health, and gaps in the literature. Our analysis considered the meaning of the results in relation to our research question and objectives, and the broader implications of the findings in terms of future research and practice in the field ( Garry *et al*., 2020).

### Consultation

This scoping review represents the work of the PART-IM (Participatory and Arts-Based Methods for Involving Migrants in Health Research) research cluster (2018–2023), Health Research Institute at the University of Limerick. The research cluster brings together arts-based and participatory scholars from medicine, nursing & midwifery, and the performing arts. The research cluster includes partners from an independent non-governmental organisation, Doras, working to promote and protect the rights of migrants through direct support, advocacy, and integration support. Through a series of research cluster meetings, NGO partners were involved in the design of the scoping review and provided feedback on the analysis and interpretation of the identified literature. They were invited to discuss preliminary findings with the aim of informing ideas about future research ( Levac *et al*., 2010).

## Results

### Search results

**Figure 1.**
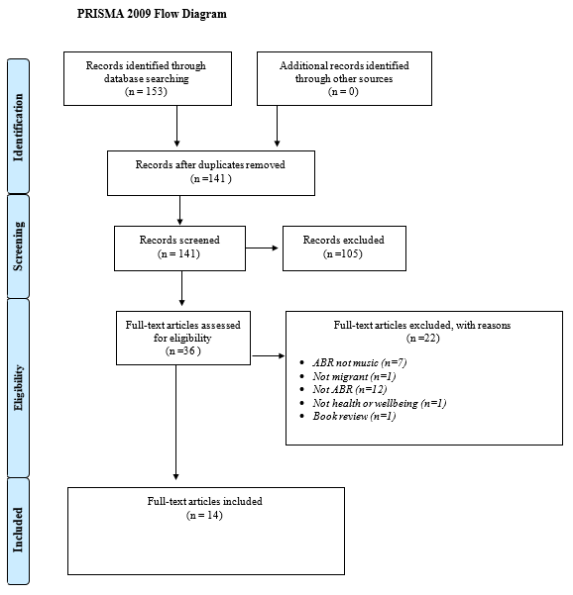
PRISMA flow diagram ( Moher *et al*., 2009).

The initial literature search yielded 153 results which were exported to Mendeley reference manager. Following the removal of duplicates, 141 articles were screened by title and abstract, and full text where necessary. 105 records were excluded. 36 publications, including journal articles, reports, books and book chapters were read in full. Of these, 14 met the inclusion criteria (see Figure 1 and Table 2). No additional relevant publications were identified through checking the reference lists of included papers.

**Table 2.** Key study characteristics.

Author(S) Year Country	Methodological Design	Aims/Purpose	Study Population and Sample Size	Health Focus
Bagley & Castro-Salazar, 2012 USA	Qualitative. Critical arts- based research	“The article reflects on how critical arts-based research in education can function as a means to legitimise, empower and promote the voices of the educationally and socially marginalised” (p.239)	“Undocumented American students of Mexican origin, living in the USA”(p.242) n=6 were interviewed. Additional artists/performers represented the interviewees’ stories through performance. Total number of participants not specified.	Social inequalities related to prejudice, discrimination and disenfranchisement. Exclusion from health care and other public services.
Bagley & Castro-Salazar, 2019 USA	Qualitative. “Arts- based inquiry, Critical Race Theory (CRT), Performance ethnography”(p.945).	“[To] reflect on the methodological issues raised by qualitative research addressing the ways in which critical arts-based research affects research participants as artists, subjects and audience” (p.945)	Mexican-Americans: Audience members (n=20), “undocumented Mexican-Americans on whose narratives the performance was based” (p.948) (n=4), artists involved in the production (n=3, 1 painter, 1 choreographer, 1 poet) All the “artists were of Mexican birth or heritage” (p.948). Total: n=27	The challenges of undocumented status: exclusion from health care and other public services. Living in fear of deportation
Blomfield & Lenette, 2018 Australia	Qualitative Analytical Autoethnography	“[To] open up broader discussions about artists’ responsibilities to produce counter-narratives that value refugees’ perspectives and voices” (p.322).	A young Somali asylum seeker in Australia	Social inequalities related to asylum seeker status. Ethical discussion as to how arts-based health and social science researchers can “fulfil their ethical obligations towards the agency and self-expression of refugees and asylum seekers” (p.327)
Blomfield & Lenette, 2019 Australia	Qualitative. Artist/ researcher critical reflexivity.	“To illustrate issues of anonymity and representation” (p.175) in documentary filmmaking.	A young Somali-Australian asylum seeker awaiting “an outcome on her refugee status determination in Australia” (p.175).	Social inequalities related to refugee/ asylum seeker detention. “lack of autonomy over basic rights […] disempowerment or disillusionment” (p.177).
Harkins *et al*., 2016 UK	Qualitative. Arts-based approaches and ethnography.	To address a deficit in research regarding the delivery and the impacts of preventative arts-based interventions aimed at reducing “mental health problems among disadvantaged children and adolescents” (p.25).	Multiple groups: “front-line intervention staff and volunteers (63); intervention management staff and board members (11); and intervention partners (52)” (p.27). Participant drawing exercise (110 children aged 6–9) Participatory filmmaking exercise: (6 children aged 12–14 years). The programme is for children from disadvantaged backgrounds, including “migrant and minority ethnic children” (p.33).	Mental and emotional wellbeing of young people in “El Sistema Scotland’s ‘Big Noise’ orchestral programme” (p.25)
Hiltunen *et al*., 2020 Finland	Qualitative. Participatory arts-based research.	“[To] identify the specific role of art in[…] negotiations of belonging” (para 3). The overall “aim is to widen the scope of research on belonging” (para 4).	Participants in the **‘**Crossing Borders Project’ **: “**recently arrived migrants and long-term residents” (para 14).	Arts, belonging and mental health
Hudson, 2016 Canada	Qualitative. Critical ethnography.	“[To] investigate the migration of indigenous youth from the reserve to the urban environment” (p.188) by investigating “the implications of an arts-based programme for youth […] as a resource for learning” (p.188)	The study focuses on the experiences of “two Indigenous young adults” (p.188) who utilised services at a native youth drop-in centre in Toronto, Canada. n=2.	Issues encountered by Indigenous youth negotiating “the dualities of belonging to ancestral place and the urban environment” (p.189) in Canada.
Lenette, 2019 Australia	Qualitative. Multiple participatory arts-based research designs.	“to explore what uses there might be for arts-based research in refugee studies and what factors to be *mindful of* when using arts-based methods” (p.xiii)	Multiple studies: people with refugee and asylum seeker backgrounds. Forced migration context.	Health and wellbeing issues in forced migration and resettlement contexts. Music and wellbeing.
Lenette *et al*., 2019 Australia	Qualitative. Collaborative arts- based methods.	The paper discusses a research-arts partnership with “a group of South Sudanese women” (p.751) who shared their “artistic and cultural practices […] during a series of creative workshops at a community gallery in Sydney” (p.751).	“A group of South Sudanese Dinka women” (p.751)	Exploring gendered experiences of resettlement: women's health related issues.
Millar & Warwick, 2019 UK	Qualitative. Exploratory and focused case study.	“[To] improve understanding of the relationship between music practice and the wellbeing of young refugees” (p.67).	“Yazidi music participants aged 11–18” (p.67). Music workshops and lessons: varying between 3 and 12 participants. Semi-structured interviews: n=6 (three boys and three girls).	Developing supporting environments for young refugees. Young people's reflections on what it means “to feel healthy in their society”, in mind and body (p.71).
Nunn, 2016 Australia	Qualitative. Collaborative, participatory arts-based research.	The article discusses a film project aimed at producing “audio-visual materials that could both contribute to existing research on refugee background youth and be disseminated to the general public as a means to increase awareness of the experiences of these young people” (p.2).	‘Somali-Australians’ (p.1), aged 13–18. Participants n=20.	Increasing awareness of young people's experiences of resettlement while fostering “artistic and psychosocial competencies” (p.2)
Nunn, 2017 Australia	Qualitative. Collaborative arts- based research.	“explores the capacity of arts-based research approaches to produce knowledge about migration generations that is difficult to capture via traditional research methods and to disseminate it beyond the academy in forms that actively contribute to intergenerational understanding in immigrant- background communities” (p.2)	Vietnamese-Australian artists and family/community members. n=22, including seven artists. Audience member feedback form: n=39.	Intergenerational relationships and communication in migration and settlement contexts. Emotional and mental health.
Nunn, 2018 UK	Qualitative. “Participatory arts- based” (p.1) research.	Dispersed Belongings’ “utilised a participatory arts-based approach to explore experiences of (non) belonging among resettled refugee young people in regional resettlement locations in Australia and the UK” (p.4).	Artist-researchers: “15–24 year old refugee- background young people” (p.7) (n=24). “Karen young people (5 female, 3 male) commenced the study in Bendigo, with five (3 female and 2 male) participating in the final presentation event. Youth artist- researchers had lived in Australia for between one and nine years, arriving aged between 7 and 20 years” (p.7) […] “Syrian and Kurdish Syrian young people (4 female, 12 male; 4 Kurdish)” (p.7) (n=16 participated at the commencement of the study with n=14 “participating in the final presentation event” (p.7).	Promoting wellbeing for young people negotiating resettlement, enabling a sense of belonging and safety. Establishing supports for young people who “can experience high levels of fear for their safety and wellbeing, which can inhibit their ability to establish belonging in local communities and activities” (p.9).
Sunderland *et al*., 2015 Australia	Qualitative. Exploratory narrative.	To explore the relevance of Schulz & Northridge’s (2004) “health equity and SDOH framework […] for both mapping and planning for sustainable health and wellbeing outcomes for participatory music programmes” (p.2)	“[F]ive refugee and asylum seeker participants involved in Scattered People” (p.3) music programme.	“Social determinants of health” (p.1). Music and wellbeing in relation to “refugees and asylum seekers'’ (p.1) experiences of detention.

## Summary of findings

### Objective 1: The extent of current publications using music in migrant health research and identifying itself as ABR

The fourteen publications identified in our review were written by a total of twenty-five authors, with a number of authors featuring in several publications. Lennette appears in five ( Blomfield & Lenette, 2018; Blomfield & Lenette, 2019; Lenette, 2019; Lenette *et al*., 2019; Sunderland *et al*., 2015) Nunn in three ( Nunn, 2016; Nunn, 2017; Nunn, 2018); and Bagley & Castro-Salazar in two ( Bagley & Castro-Salazar, 2012; Bagley & Castro-Salazar, 2019). While some of the publications are single authored pieces ( Hudson, 2016; Lenette, 2019; Nunn, 2016; Nunn, 2017; Nunn, 2018), all of the studies report on collaborative and/or participatory arts-based research projects with multidisciplinary ( Hiltunen *et al*., 2020) and interdisciplinary ( Harkins *et al*., 2016; Sunderland *et al*., 2015) teams. These include academics in collaboration with artists and participants from a variety of disciplinary and artistic backgrounds. In some cases, these roles are blended in the form of an artist-scholar ( Millar & Warwick, 2019; Sunderland *et al*., 2015). Other studies were co-authored by artists and academics ( Blomfield & Lenette, 2018; Blomfield & Lenette, 2019; Lenette *et al*., 2019) or between academics working in collaboration with artists from the specific migrant community in the study ( Bagley & Castro-Salazar, 2012; Bagley & Castro-Salazar, 2019; Nunn, 2017). Several of the studies were in partnership with art galleries, multicultural centres or other community-based organisations ( Bagley & Castro-Salazar, 2012; Bagley & Castro-Salazar, 2019; Harkins *et al*., 2016; Hiltunen *et al*., 2020; Hudson, 2016; Lenette, 2019; Nunn, 2016; Nunn, 2017; Nunn, 2018). Disciplines included music, humanities, health and human services ( Sunderland *et al*., 2015), public health research and policy, and evaluation of the impact of community-based programmes ( Harkins *et al*., 2016). Settings included refugee and asylum seeker detention centres ( Blomfield & Lenette, 2018; Blomfield & Lenette, 2019), indoor and outdoor community locations and people’s homes ( Blomfield & Lenette, 2018; Lenette, 2019; Nunn, 2016; Sunderland *et al*., 2015).

***Migrant populations.*** Twelve of the publications discuss arts-based research projects exclusively involving migrant people. None of the authors provided a source reference to define the migrant population that they were working with, e.g. the UNHCR definition for refugee. Participants in the studies are described as undocumented Mexican-Americans and artists of Mexican birth or heritage ( Bagley & Castro-Salazar, 2012; Bagley & Castro-Salazar, 2019); first, 1.5, and second generation Vietnamese-Australians, including artists and their family members ( Nunn, 2017); refugee-background young people, including Karen young people in Bendigo, Australia, and Syrian and Kurdish Syrian young people in Gateshead, UK ( Nunn, 2018); a young Somali asylum seeker woman ( Blomfield & Lenette, 2018; Blomfield & Lenette, 2019); Somali-Australians, aged 13–18, described as the first generation of young people growing up in a Somali-Australian immigrant community ( Nunn, 2016); Indigenous young adults in the context of migration from the reserve to the urban environment in Canada ( Hudson, 2016); a group of South Sudanese Dinka women, mainly mothers and daughters, who had been living in Australia for 10–15 years ( Lenette *et al*., 2019); refugee and asylum seeker members of the Brisbane-based Scattered People music initiative, including two Iranian women, a Kurdish male and female married couple, and one Sri Lankan male, aged between 24 and 50 years of age ( Sunderland *et al*., 2015); and Yazidi young people, aged 11–18, from Iraq and Syria living in a camp in northern Greece ( Millar & Warwick, 2019). Lenette’s (2019) book discusses multiple groups and refers, throughout, to “people with lived experiences of forced migration as Knowledge Holders” (p.12). The remaining two studies describe participants as recently arrived migrants and long-term residents ( Hiltunen *et al*., 2020), and participants in El Sistema Scotland’s Big Noise music programme for children from disadvantaged backgrounds, including “migrant and minority ethnic children” ( Harkins *et al*., 2016, p.33).

***Defining arts-based research*.** While all studies self-identified as ABR, most of the publications assume an implicit understanding of ABR, focusing more on why one might use this approach rather than how it is defined. Lenette (2019) describes an ABR approach as “the use of any art form at any point in the research process, to generate, interpret, or communicate new knowledge” (p.27). She refers to McNiff (2008) to provide a definition of arts-based research as: “[The] systematic use of the artistic process, the actual making of artistic expressions in all of the different forms of the arts, as a primary way of understanding and examining experience by both researchers and the people that they involve in their studies” ( McNiff, 2008 cited in Lenette, 2019 p. 28). Bagley & Castro-Salazar (2012) view a critical arts-based approach as a means to address social inequities related to prejudice, discrimination and disenfranchisement. Blomfield & Lenette (2018) use critical reflexive narratives to “open up broader discussions about artists’ responsibilities to produce counter-narratives that value refugees’ perspectives and voices” (p.322). Nunn (2016) uses a collaborative participatory arts-based approach in a film project aimed at increasing awareness of the experiences of first generation young people growing up in a Somali-Australian immigrant community. Two studies use a participatory arts-based approach to study the concept of belonging and (non)belonging ( Hiltunen *et al*., 2020; Nunn, 2018). Nunn (2017) uses collaborative arts-based research as a means to produce and communicate new knowledge about migration generations ( Nunn, 2017).

***Health context*.** Our review did not yield any findings of music in ABR for migrant health research which had explicit clinical aims, or which took place primarily in a clinical setting. Nonetheless all 14 studies reflected socio-cultural aspects of health and wellbeing, and highlight the complexity and multi-dimensionality of negative and positive wellbeing from the perspectives of the participants. Studies focusing explicitly on music, health and wellbeing show that lived experiences of health and wellbeing are context specific and dependent on multiple factors ( Harkins *et al*., 2016; Millar & Warwick, 2019; Sunderland *et al.*, 2015). The development of confidence and self-esteem through enhancing musical expertise was “seen as especially relevant for migrant and minority ethnic children who struggled to learn in an English language environment” ( Harkins *et al.*, 2016). Sunderland *et al*. (2015) explored the impact of engagement in the Scattered People music initiative with refugee and asylum seeker background people using Schulz and Northridge’s (2004) health and wellbeing conceptual framework. In their study, wellbeing outcomes self-reported by participants generated themes that did not fit into the existing framework for understanding wellbeing. These are: “cultural expression, music making, and social identity” (p.3). Millar and Warwick (2019) explore musical engagement as a means to develop supports for young refugees in the context of protracted displacement through strengthening supportive environments, community action, and personal skill development.

Two studies focus on social justice issues associated with undocumented status in U.S., such as exclusion from health and other services ( Bagley & Castro-Salazar, 2012; Bagley & Castro-Salazar, 2019). Nunn (2018) explores how young people negotiate resettlement, and notes the importance of generating and communicating understandings of those experiences through arts-based research in order to develop support networks in response. Lenette *et al*. (2019) focus on the experiences of women from South Sudanese backgrounds in an Australian resettlement context and note the importance of contributing “gender-specific and strengths-based perspectives to the literature on impacts of community-based arts initiatives” (p.757). Lenette notes that studies on health from a biomedical perspective are prolific in refugee research due to the many trauma related issues people face in the context of forced migration. She cautions that a disproportionate focus on these issues has often overshadowed the equally-important need to understand “sociocultural, ‘bottom-up’ perspectives, and the intersectionality of issues linked to wellbeing” ( Lenette, 2019, p.7).

### Objective 2: The research stages (participating, generating data, interpreting, disseminating) in which music features as an ABR method or part thereof

We extracted information about the musical elements and the role of music for data generation, interpretation and analysis from the research process descriptions in the papers.

***Participation*.** The most significant role played by music as an arts-based method in all the studies is as a tool of participation, supporting sustained recruitment, and active creative and dialogic engagement in the research process.

While the exact nature or role of the musical element is not always clear, several of the studies are based on periods of participative engagement. In Harkins *et al.* (2016), a group of children worked with a filmmaker to make a (participant-led) documentary with the pre-set aim of capturing their experiences of participation in the music programme. Hudson (2016) based her research on a 12-week programme in which indigenous youths participated in arts-based workshops and field trips. In Nunn (2018) data were collected through 8–12 arts/music sessions over a period of three months. Millar and Warwick (2019) collected data over a five week period during which the young Yazidi music participants, resident in a refugee camp in northern Greece, were invited to participate in individual and group music lessons and workshops. In Hiltunen *et al.* (2020) researchers collaborated with practitioners and participants in three types of collaborative workshops over a period of three to four months. Lenette *et al*. (2019) highlight the importance of facilitating a culturally safe, women-only space in a workshop series for resettled South Sudanese Dinka women. Participatory music projects particularly created spaces of support for young people to reflect on their experiences and feelings ( Harkins *et al*., 2016; Millar & Warwick, 2019; Nunn, 2018).

***Data generation*.** Music is mainly discussed in terms of its role in generating knowledge through lived experiences of active engagement. Song performances generate knowledge by connecting people at an emotional level, encouraging empathetic responses, and creating a space for sharing experiences and stories ( Lenette *et al*., 2019; Millar & Warwick, 2019; Sunderland *et al.*, 2015). Collaboratively written lyrics constitute an important source of data and a means to represent lived experience ( Lenette, 2019; Nunn, 2018). Well known pop songs can take on new meanings in the context of forced displacement, thereby communicating important messages relating to issues of social justice and the plight of detained refugee and asylum seeker people ( Lenette, 2019). The opportunity to sing in a first or familiar language helps people to articulate feelings of pride in their heritage, while also expressing new and shared identities ( Sunderland *et al*., 2015). Participant-observation is recognised as a key form of data generation where music facilitators/researchers immersed in the field can access knowledge by co-participating at the site of engagement, and sharing insights from practice ( Millar & Warwick, 2019; Nunn, 2018). This is particularly insightful where community musicians can gain access to spaces, such as asylum seeker detention centres, sometimes inaccessible to researchers ( Lenette, 2019).

***Interpretation*.** The role of music as a tool for data interpretation is not widely discussed in the literature. Nunn (2017) notes that there were some gaps in understanding regarding the artistic representations of interview data, including karaoke, in “Translations-Generations”. She argues that non-literal research representations challenge audience members to interpret the work. While this is one of the benefits of artistic representations, it does “provoke consideration of the potential for arts-based representations to be as exclusionary as academic ones” (p. 13). Bagley & Castro-Salazar (2012) mention that the artists, including a musician, were given a free hand to interpret the interview data. Blomfield & Lenette (2019) explain the intent behind the use of instrumental background music in their documentary film project. Its purpose is to foreground the verbal narrative of the young female asylum seeker.

***Dissemination*.** In the studies focusing exclusively on music, the performance of songs and instrumental music are the most common forms of dissemination. The Scattered People ( Lenette, 2019; Sunderland *et al*., 2015) disseminate their music, and their political messages, through performance and recordings. Lenette (2019) notes that “singing is one of the most effective ways of disseminating such [political] messages to wide audiences” (p.184). In Sunderland *et al*. (2015) “[P]articipants felt that their music gatherings and performances had the potential to improve understandings of refugee experiences, and hopefully lead to more acceptance”. (p.10). Harkins *et al*. (2016) provide a link to the documentary film made by the young people, and this enables the reader/viewer to experience the children’s orchestral programme first-hand. Background music is used in film ( Blomfield & Lenette, 2019; Nunn, 2016), and as pre-performance music at a live multi-arts event that included research-based post-performance karaoke for audience members ( Nunn, 2017). Other multi-arts events drew on music, spoken word, photography, installation, painting and digital media “reflecting the complexities of refugee lives” ( Nunn, 2018, p.32).

### Objective 3: Identify whether particular musical practices are used more than others as part of the research design and implementation

Only three studies focus exclusively on music ( Harkins *et al.*, 2016; Millar & Warwick, 2019; Sunderland *et al*., 2015). Lenette (2019) includes a chapter on community music, focusing mainly on the Brisbane-based Scattered People music initiative. Lenette *et al*. (2019) and Nunn (2017); Nunn (2018) discuss music alongside other art forms. In the remaining studies, the role of music is less prominent, or is unclear. Singing is the most highly reported musical activity ( Harkins *et al*., 2016; Lenette, 2019; Lenette *et al*., 2019; Millar & Warwick, 2019; Nunn, 2017; Nunn, 2018; Sunderland *et al.*, 2015), followed by songwriting ( Lenette, 2019; Lenette *et al*., 2019; Millar & Warwick, 2019; Nunn, 2018; Sunderland *et al*., 2015), background music in documentary filmmaking ( Nunn, 2016; Blomfield & Lenette, 2019) and orchestral music ( Harkins *et al*., 2016). Bagley & Castro-Salazar (2012); Bagley & Castro-Salazar (2019) mention that one of the collaborating artists was a musician, but the performance examples discussed in the articles do not include the musical element. Hudson (2016) mentions music as part of a dataset that emerged from an arts-based programme for a number of young people, but the article focuses on two indigenous youths and on photography. Hiltunen *et al.* (2020) mention exploring sound as an expressive tool and listening to “the rhythms, tones and silences in each other’s languages” (para. 23). They also mention “belonging through music” (para. 22) as one of several film themes in the filmmaking workshops, and “sound and video poems” (para. 25) as artistic outputs from creative writing workshops. However, there is no further elaboration on these examples. In one study, singing and dancing were not included in the research design but emerged spontaneously in a participant-led project with a group of South Sudanese Dinka women in Australia. The knowledge generated through the shared musical engagement was then incorporated into the research ( Lenette *et al.*, 2019).

### Objective 4: Identify the key strengths and challenges discussed in the literature around the use of music as a research tool in migrant health research

While strengths, challenges and ethical considerations were not always explicitly named as such by the authors, the following points can be extrapolated based on their project descriptions.

***Strengths*.** Lenette (2019) notes that ABR “integrates artistic practice with research processes as a crucial way to understand lived experiences” (p.27), and challenges “dominant research models that may fail to create culturally safe research spaces” (p.31). She highlights a number of positives that emerge from ABR into musical engagement in the context of refugee and asylum seeker experiences of detention in Australia. These include music’s capacity to reveal knowledge related to health and wellbeing; to provide a sense of comfort and security; to help maintain cultural songs and practices; and to connect people from different cultures with each other and the wider community. Music has the capacity to provide an escape from daily realities, and can help to alleviate “stress, loneliness, uncertainty and hopelessness” (p.195). Millar & Warwick (2019) found that “activities involving music practice can impact positively on young people’s wellbeing, enabling the development of emotional expression, improved social relations, self-knowledge, and positive self-identification” (p.67). Nunn (2017) argues that karaoke is a highly appropriate form which facilitated embodied affective engagement with the research data. Sunderland *et al.* (2015) note the capacity of arts-based music research to add new culturally relevant categories to existing health and wellbeing frameworks.

***Challenges*.** Nunn (2018) refers to inherent risks in participatory arts projects in general. In the “Dispersed Belongings” project, not all experiences of the research presentation events were positive. Some people were nervous about performing, while one Syrian woman and her family experienced criticism over her involvement in the performance. In addition, she lost trust in the research project team as they could not prevent Syrian audience members from videoing the performance (p.36). Nunn (2017) explains that the inclusion of non-text based artworks and the use of the Vietnamese language designed to make the work more accessible to non-English speaking first generation Vietnamese Australians “was not as successful as it might have been in facilitating an ethical and accessible mode of dissemination” (p.13) due to challenges experienced by some audience members in interpreting the artistic work. Sunderland *et al*. (2015) specify the small number of participants as a limitation but note that rich data emerged from the collaboration, suggesting that future research could draw comparisons to studies with marginalised groups who are not engaged in musical activities. Similarly, Harkins *et al*. (2016) suggest that, while qualitative methods suited the study focus, the lack of a validated wellbeing measurement framework limits the potential for comparison with other studies.

***Ethical considerations*.** There were no specific ethical issues related to any one art form, with the exception of awareness of the editing process as a political act in filmmaking ( Blomfield & Lenette, 2018). Lenette (2019) draws attention to ethical issues such as “reciprocity, power differentials, and assumptions of ‘vulnerability’ and ‘anonymity’” (p.86). While she emphasises the importance of using a trauma-informed approach which is sensitive to participants’ past circumstances, she argues that “the determination of ‘vulnerability’ should be contextual and project-specific” (p.91). Two studies note the importance of incorporating a flexible ethical framework “to avoid latent harm within the research process” ( Millar & Warwick, 2019, p.72), and as a means to “engage refugee participants in meaningful ways” ( Sunderland *et al*., 2015, p.6). Other ethical considerations relate to decisions around the use of photography ( Hiltunen *et al*., 2020), valuing diverse experience and knowledge ( Lenette *et al.*, 2019), and adopting ethical guidelines specifically relevant to indigenous communities ( Hudson, 2016). Blomfield & Lenette (2018) focus exclusively on the ethical considerations for artists engaging in collaborative work with refugees and asylum seekers. While ABR researchers advocate for the role of art in creating counter-narratives and challenging negative perceptions of migrants, they caution against any simplistically positive depiction of art and artists in the ABR process, noting that artists are not immune to the perpetuation of detrimental tropes around the people their work attempts to ‘represent’ ( Blomfield & Lenette, 2018).

## Discussion

The purpose of this scoping review was to ascertain what is known about the use of music as an arts-based method in migrant health research. In doing so, our goal was to advance knowledge regarding the use of creative methods and tools supporting migrant participation in meaningful partnerships in health research.

The findings demonstrate a concentration of publications in journals related to methodology, education, health and culture, but not with a specialist focus on music. Rather than providing a specific definition of ABR, authors most often described an arts-based approach, based on their own previous work or other published studies. ABR is evident across all stages of the research cycle. Music as ABR facilitated recruitment, active participation in data generation, and dissemination.

While this review builds on the work of several publications and reviews regarding ABR and health ( Boydell *et al*., 2012; Boydell *et al*., 2016; Coemans & Hannes, 2017; Fraser & al Sayah, 2011) we found that the literature on *the specifics of using music in migrant health and wellbeing research and identifying itself as ABR is modest*. Yet it is highly informative in terms of current practices, strengths and challenges as well as about the importance of researcher reflexivity. The 14 identified references are concentrated on populations of asylum seekers, refugees, resettled immigrants, and undocumented migrants. This reflects the broad pattern in the field of migrant health research: these groups are heavily researched due to their complex physical and psychological health needs ( Sweileh *et al*., 2018). Like many studies in the field, the authors did not provide a source reference to define the migrant population that they were working with ( Hannigan *et al*., 2016). Authors did provide detailed descriptions of the participants in their studies and this may be because of the specific situated, contextual focus of ABR and its attention to bringing individual stories, histories, and contexts to the fore ( Leavy, 2009).

The studies provide important insights into how music is being used in ABR in the context of lived experiences of migration *.* Music is primarily used as a means to co-produce and communicate the embodied knowledge of participants’ lived experiences of migration through singing, songwriting, participatory music-making, background music in film, mixed media, and multi-arts events. The findings reveal several positive outcomes around using music in ABR in migrant health including the ability of music to communicate non-verbally; to contribute to a sense of comfort; to both maintain and expand cultural knowledge and traditions; to provide an escape from difficult realities and contribute to psychological healing; and to enhance emotional expressivity, self-identity and social relationships. The findings point to both the ways in which music and singing can be used to create supportive participatory spaces for research and the importance of skilled facilitation to hold space safely and effectively as the embodied knowledge and emotional expressions come to the fore.

While there are some challenges and ethical issues with ABR identified in this review, overall the literature points to strengths that are characteristic of music in intercultural community projects in terms of social bonding and positive personal and community outcomes ( Balsnes, 2016; Howell, 2018; Marsh, 2019; Phelan, 2017; Raanaas *et al*., 2019). The documented strengths of ABR using music resonate with the published literature about how singing, in particular, can help to foster participation, social connection and a sense of community ( Ahlquist, 2006; Balsnes, 2016; Bithell, 2014; Pearce *et al*., 2015; Phelan, 2017).

### Limitations

We acknowledge the relatively small number of results in this review, which points to a lack of research in this area. This presents challenges in terms of making comparisons with other research and research methodologies in the field. We have been monitoring new publications since 2020 and there are few ( Nunn, 2022; Wells *et al*., 2020(first published online in Dec. 2020)). These studies wholly reflect the findings and analysis presented here. This low level of publications in the three years is reflective of the field and our findings. Studies were confined to the English language and limited to publication dates between 2009 and 2020. Publications about music projects that may align with the concept of ABR but do not self-identify as ABR have not been included.

### Recommendations for future research

There is evidence that there is a positive impact of music as ABR in migrant health research. However, the identified limitations and gaps in these publications form the basis of our four key recommendations:

**(1) Increased detail on the musical element in ABR**

In order to fully assess the value of music as ABR, it is necessary to have detailed information on the musical element itself. Additional detail concerning musical decision making processes, repertoire, musical forces, and examples of lyrics, as well as a discussion on the creative process of selecting, composing, rehearsing and performing would help shed light on the dynamics of musical practice in the context of migrant health research.

**(2) Explicit identification of research as ABR**

While our focus was on music as an arts-based research method, our search did not yield any publications in music-specific journals. Even a cursory examination of publications related to participatory music practices with migrants in both clinical and community settings show significant evidence of research which broadly fits Leavy’s (2009) definition of ABR quoted earlier, as the use of any art form at any point in the research process. Lenette (2019) specifically identifies community music, for example, as a form of ABR. Nonetheless, many of these musical practices and publications do not themselves use the terminology of ABR.

While it would be easy to dismiss this as a point of nomenclature, it presents significant issues of access for interdisciplinary researchers who may easily miss such publications, if they cannot identify them through a keyword search that includes arts-based research/ABR. Where ABR is used, its explicit identification will facilitate the inclusion of ABR publications which focus on music with other, more visible ABR publications in film, theatre, creative writing and photography.

**(3) Greater recognition of a multi-arts understanding of music in the context of ABR**

While our search focused on music as an arts-based research method, the results yielded mostly multi-arts projects within which music was one of several artistic practices.

In participatory, multi-arts experiences, it is recognised that artistic practices rarely exist in isolation. Understanding music within a wider multi-arts context allows for a holistic engagement with not only the sonic dimensions of experience, but also the gestural, tactile, poetic and visual. This whole person engagement supports migrant health research that is sensitised to the need for anti-oppressive research methods in contexts such as post-conflict trauma.

**(4) Harnessing the participatory potential of ABR using music and singing across the research cycle to increase migrant involvement in health research**

The identified studies demonstrate that music in ABR is most common for recruitment and engagement but that it can be used in all aspects of the research cycle. Music was evident in data generation, interpretation and dissemination. Thus ABR using music and singing has a role to play in increasing migrant participation in health research and encouraging researchers to think more creatively about the tools and material practices ( Kothari, 2001) that they use in and for the co-design of participatory migrant health research projects.

## Conclusion

Arts-based research using music shows promise for capturing the complexity of migrants’ lives and health issues in an ethical way. Thus, the findings of this review support the idea that the benefits of music and singing in intercultural projects may be harnessed for migrant health research. Further, ABR using music and singing has specific sensory features that may offer advantages over other methods for involving migrants in health research, and thus warrant further investigation in empirical studies about its processes and impacts in multiple clinical and community settings and with a variety of migrant groups.

## Data Availability

All data underlying the results are available as part of the article and no additional source data are required. Garry, Fran; MacFarlane, Anne; Murphy Tighe, Sylvia; Punch, Pattie; Phelan, Helen (2023). Underlying Data for 'A Scoping Review of the Use of Music as an Arts-Based Method in Migrant Health Research'. University of Limerick. Dataset. https://doi.org/10.34961/researchrepository-ul.24201423.v2 ( Garry *et al.*, 2023). This project contains the following extended data: Full Search Strategy for ScR of the Use of Music as an Arts-Based Method in Migrant Health Research.pdf Full Details of Included Studies for ScR of the Use of Music as an Arts-Based Method in Migrant Health Research.xlsx Summary Table of Musical Elements in ABR for ScR of the Use of Music as an Arts-Based Method in Migrant Health Research.pdf Figshare: PRISMA-ScR checklist for 'A Scoping Review of the Use of Music as an Arts-Based Method in Migrant Health Research'. https://doi.org/10.34961/researchrepository-ul.24201423.v2 ( Garry *et al.*, 2023). Data are available under the terms of the Creative Commons Attribution 4.0 International license (CC-BY 4.0).
