# Supplementary material for: A scoping review of the use of music as an arts-based method in migrant health research
Source: HRB Open Res. 2023 Dec 6;6:74. [Version 1] doi: 10.12688/hrbopenres.13817.1 (PMC10980858; doi:10.12688/hrbopenres.13817.1)
Supplement: Peer review history

# Reviewer report: Reviewer response for version 1

**DOI:** 10.21956/hrbopenres.15124.r38021

**Published:** 2024-03-29

**Authors:** Nicola De Martini Ugolotti

**Affiliations:** 1 Department of Sport and Event Management, Bournemouth University, Poole, England, UK

This scoping review examined literature around the use of music as an arts-based research (ABR) method to extend knowledge of creative methods and tools used for migrant participation in health research.

 The inter-disciplinary team of authors selected 14 studies based on selection criteria including use and engagement with arts-based research, forms of music, details of migrant groups, study teams, ethical issues, health focus, and evaluation of music-oriented interventions and research designs.

 The scoping review offered a justification for its specific scope on music and arts-based methods in relation to migration and health, and overall offered a useful overview of how music and arts-based methods have been used in research projects with people with (forced) migration backgrounds and in relation to health research. The clear assessment of key themes, trends, methodological engagement with music, as well as strengths, challenges and ethical considerations makes this scoping review a useful resource for academics, practitioners and policy-makers working at the intersection of migration, arts-based research, and health.

 That said, I consider relevant to offer a few points as constructive challenges/considerations to the authors regarding how researchers might find useful to engage with already-existing music spaces while conducting arts-based and participatory research with people with (forced) migration background. Such considerations can be seen particularly relevant and connected to the authors’ points relating to the need to understand “sociocultural, *‘bottom-up’ perspectives*, and the intersectionality of issues linked to wellbeing” (Lenette C, 2019, [Ref 2] p.7 as cited in the scoping review, emphasis added) through and from the different registers, practices, and domains of music.

 The first point relates to the relevance of considering music beside the establishment of arts-based research-design and interventions by researchers. That is, I invite the authors to consider in further research the relevance of music not only within the remit of ad-hoc, time-limited, arts-based research intervention. Research conducted in already-existing and often community-based musical spaces created by people with (forced) migration backgrounds have highlighted how music can constitute a register of experience that can highlight insights, experiences and forms of knowledge relating, but not limited to health and well-being among diverse (forced) migrant communities (see Western T, 2020 [Ref 4]; Pistrick E, 2020 [Ref 3]; De Martini Ugolotti N, 2022 [Ref 1], online first, July 2020; Wilcock C, 2023 [Ref 5]).

 While I appreciate the authors’ justification to delimit the scope of this specific manuscript, I would invite the authors to consider also how only focusing on researchers-initiated music-methods and interventions could obscure spontaneous musical domains that can reveal the connections between lived migration trajectories, overarching socio-political processes (e.g. migration policies), and daily practices of sociality, health, well-being and (self)care.

 Building on the previous point, while the spatial settings for art-based research are briefly mentioned in the scoping review (in “Summary of findings, Objective 1”), I consider useful paying more attention to *where music takes place* as an important element that influences the dynamics enabling and shaping music experiences and their associations with health and well-being. That is, it matters if a music group or arts-based research takes place in a detention or reception centre, in a community space or in a public park or square, as all these spaces materialise and shape the possibilities, benefits and challenges surrounding the potentialities of music-based foci and practices in research and health domains (as discussed by Western T, 2020 [Ref 4]; Pistrick E, 2020 [Ref 3]; De Martini Ugolotti N, 2022 [Ref 1]).

 Finally, a focus on musicking beside and beyond music as an arts-based methodology can also contribute to critically interrogate some of the associations between practices of research dissemination through music as forms of empowerment, and/or ways of social and public participation (as briefly mentioned in the “Challenges” section of the scoping review). Pistrick’s (2020) work showed how music-making in the context of a refugee reception camp in Germany was seen as relevant by social workers only when relating to a “desire” to participate in society (e.g., through or in preparation for public performances), while more leisurely and unstructured musicking in informal, everyday spaces could be perceived as irrelevant or “disturbing noise”.

 Linking the insights from some of the publications included in the scoping review to these points, it is fundamental to acknowledge how understandings of public music performances as engagement “pinnacles” of arts-based research might not always align with the experiences, meanings, and concerns of participants with (forced) migration background (see also De Martini Ugolotti N, 2022 [Ref 1]). 

 Overall, as a researcher who conducted studies in relation to music and (forced) migration, I commend the authors’ synthesis of key themes and challenges in relation to the specific scope of this review.

 Concurrently, I also invite the authors to consider and engage with musical spaces and domains that exist beside and beyond those initiated by researchers, as sites that can “generate, interpret, or communicate new knowledge” (Lenette C, 2019, [Ref 2] p. 27) on the nexus between diverse (forced) migration experiences and trajectories, health, and wellbeing across different national, political, and social contexts.

Are the rationale for, and objectives of, the Systematic Review clearly stated?

Yes

Is the statistical analysis and its interpretation appropriate?

Not applicable

Are sufficient details of the methods and analysis provided to allow replication by others?

Yes

Are the conclusions drawn adequately supported by the results presented in the review?

Yes

Reviewer Expertise:

Music, leisure, physical culture, migration and forced migration, urban and cultural geographies

I confirm that I have read this submission and believe that I have an appropriate level of expertise to confirm that it is of an acceptable scientific standard.

## References

[1] : Music-making and forced migrants’ affective practices of diasporic belonging. *Journal of Ethnic and Migration Studies* .2022;48(1) : 10.1080/1369183X.2020.1790344 92-109 10.1080/1369183X.2020.1790344

[2] : Arts-Based Methods in Refugee Research.2019; 10.1007/978-981-13-8008-2 10.1007/978-981-13-8008-2

[3] : Dangerous fields: Existentiality, humanity and musical creativity in German refugee camps. *Violence: An International Journal* .2020;1(2) : 10.1177/2633002420949551 332-353 10.1177/2633002420949551

[4] : Listening with Displacement. *Migration and Society* .2020;3(1) : 10.3167/arms.2020.030128 194-309 10.3167/arms.2020.030128

[5] : In the groove and in the moment: epistemology and ethics in ethnography with Sudanese musician revolutionaries. *Qualitative Research* .2023;23(3) : 10.1177/14687941211047720 746-763 10.1177/14687941211047720

# Reviewer report: Reviewer response for version 1

**DOI:** 10.21956/hrbopenres.15124.r37426

**Published:** 2024-02-16

**Authors:** Kari Veblen

**Affiliations:** 1 Don Wright Faculty of Music, University of Western Ontario, London, ON, Canada

This literature review offers useful insights into community health and well-being using music with refugees, asylum seekers and immigrant populations. The goal of this study is to advance knowledge into uses of arts-based research supporting migrant participation in meaningful partnerships in health research. 

 An interdisciplinary team selected 14 from a pool of 153 studies. Criteria for selection included definitions of arts-based research, forms of music, details of migrant groups, study teams, ethical issues, health focus, and evaluation of music, among other considerations.

 Well written and easy to follow, this paper is illustrated with tables, a methodological diagram, and charts of definitions.  The 14 surveyed studies are presented in brief.  They represent a spectrum of populations and contexts from Somali-Australian immigrants, participants in El Sistema Scotland’s Big Noise music programme for children from disadvantaged backgrounds, to indigenous youth in urban Canadian settings.

 The authors note that while the exact nature or role of the musical element is not always clear, several studies rely on the participatory engagement afforded through this expressive medium. They write that the music is most significant as: “a tool of participation, supporting sustained recruitment, and active creative dialogic engagement in the research process.”

 Findings indicate that researchers employ a variety of arts-based methods, some defined, some implied.  While the studies surveyed here are modest in number, they offer rich insights into ways music is used in participant’s lived experiences of immigration.  The authors also offer recommendations for future research.

Are the rationale for, and objectives of, the Systematic Review clearly stated?

Yes

Is the statistical analysis and its interpretation appropriate?

Yes

Are sufficient details of the methods and analysis provided to allow replication by others?

Yes

Are the conclusions drawn adequately supported by the results presented in the review?

Yes

Reviewer Expertise:

music education, community music, arts based research, learning music through play, social media and music learning, sociology

I confirm that I have read this submission and believe that I have an appropriate level of expertise to confirm that it is of an acceptable scientific standard.
